# The influence of the copy number of invader on the fate of bacterial host cells in the antiviral defense by CRISPR-Cas10 DNases

**DOI:** 10.1016/j.engmic.2023.100102

**Published:** 2023-06-24

**Authors:** Zhenxiao Yu, Jianan Xu, Yan Zhang, Qunxin She

**Affiliations:** a CRISPR and Archaea Biology Research Center, State Key Laboratory of Microbial Technology and Microbial Technology Institute, Shandong University, Qingdao 266237, China; b College of Food and Biological Engineering, Henan University of Animal Husbandry and Economy, Zhengzhou 450000, China

**Keywords:** Type III CRISPR systems, Target RNA-activated Cas10 DNase, Invader copy number, Plasmid interference assay, Abortive infection, Invader clearance

## Abstract

Type III CRISPR-Cas10 systems employ multiple immune activities to defend their hosts against invasion from mobile genetic elements (MGEs), including DNase and cyclic oligoadenylates (cOA) synthesis both of which are hosted by the type-specific protein Cas10. Extensive investigations conducted for the activation of Cas accessory proteins by cOAs have revealed their functions in the type III immunity, but the function of the Cas10 DNase in the same process remains elusive. Here, *Lactobacillus delbrueckii* subsp. *Bulgaricus* type III-A (Ld) Csm system, a type III CRISPR system that solely relies on its Cas10 DNase for providing immunity, was employed as a model to investigate the DNase function. Interference assay was conducted in *Escherichia coli* using two plasmids: pCas carrying the LdCsm system and pTarget producing target RNAs. The former functioned as a de facto “CRISPR host element” while the latter, mimicking an invading MGE. We found that, upon induction of immune responses, the fate of each genetic element was determined by their copy numbers: plasmid of a low copy number was selectively eliminated from the *E. coli* cells regardless whether it represents a de facto CRISPR host or an invader. Together, we reveal, for the first time, that the immune mechanisms of Cas10 DNases are of two folds: the DNase activity is capable of removing low-copy invaders from infected cells, but it also leads to abortive infection when the invader copy number is high.

## Graphical abstract

**Figure:**
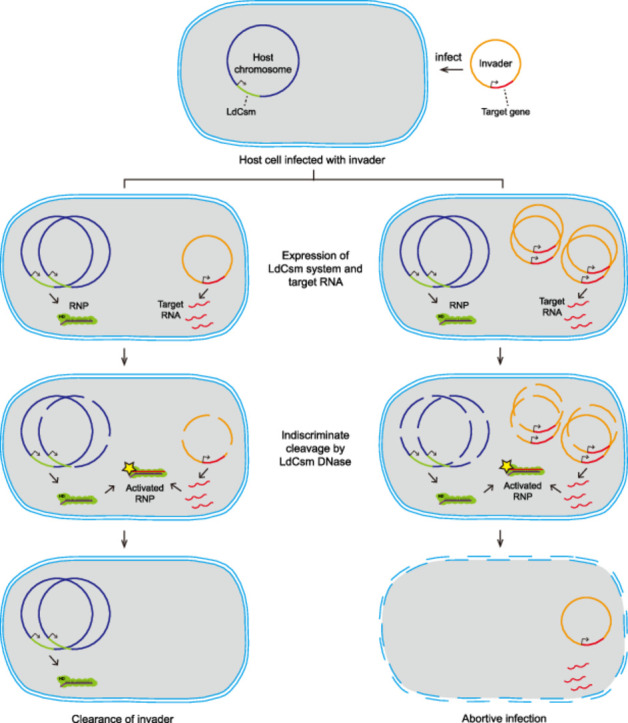

## 1 Introduction

Clustered Regularly Interspaced Palindromic Repeats (CRISPR) and CRISPR-associated (CRISPR-Cas) systems provide the adaptive antiviral defense mechanism in bacteria and archaea [1], [2], [3]. The system codes for ribonucleoprotein (RNP) effectors composed of Cas proteins and CRISPR (cr)RNAs, which are generated from expression of *cas* gene modules and CRISPR arrays, respectively. These immune systems fall into 6 different types among which Type I, III, and IV employ multiple different protein subunits to build up RNPs whereas Type II, V and VI rely on a multidomain protein to make the effector [4].

Type III CRISPR systems represent the most complicated type of the prokaryotic immunity with Cas10 as their type-specific protein (also called CRISPR-Cas10 systems). Two subtypes, III-A Csm and III-B Cmr, have been extensively characterized *in vivo* and *in vitro* [5], [6], [7], [8], [9]. Their Cas10 proteins, named Csm1 and Cmr2 individually, constitute the largest subunit in their respective effector complexes. Most CRISPR-Cas10 systems exhibit three distinctive activities, including two Cas10-hosted activities to be activated by target RNAs, i.e., the HD domain-mediated unspecific ssDNA cleavage [10], [11], [12], [13] and Palm-mediated synthesis of cyclic oligoadenylates (cOAs), and one provided by the large backbone subunit Csm3/Cmr4, namely target RNA cleavage at an interval of 6 nt [14], [15], [16], [17], [18]. Target RNAs are of two different types: cognate target RNA (CTR) and noncognate target RNA (NTR): While CTR exhibits mismatches between the 3′-flanking protospacer sequence (3′-FPS) of target RNA and the 5′-repeat handle (5′-tag) of crRNA, NTR possesses the fully complementary sequences between the two RNAs including the 3′-FPS of target RNAs and the 5′-tag of crRNAs; only CTRs have the capability to activate the two Cas10 activities [8,19,20]. In addition, target RNA cleavage by the backbone nucleases inactivates the Cas10 activities, thereby completing activation/inactivation cycle of the type III immunity [12]. Furthermore, cOAs function as a secondary signal to activate unspecific ssRNA/DNA cleavage by ancillary Cas proteins named CARF (CRISPR-associated Rossman Fold) proteins such as Csm6/Csx1, Can2, Card1, resulting in cell death or cell dormancy to host cells [21], [22], [23], [24], [25], [26], [27], [28], [29].

Genetic studies have revealed that the cOA signal transduction pathway plays a major role in mediating antiviral defense in most known CRISPR-Cas10 systems whereas the function of CRISPR-Cas10 DNases in type III immunity remains to be demonstrated [30], [31], [32], [33]. At present, both DNase and cOA signaling pathway have been implicated in successful defense of invasion by MGEs [13,20,34,35]. But most type III CRISPR systems rely on signal transduction pathways to activate cOA nuclease effectors for indiscriminate RNA cleavage, leading to cell dormancy whereas CRISPR-Cas10 DNases were implicated in the eventual target DNA clearance [36,37]. The involved mechanism was attributed to the selective degradation of transcription bubbles of DNA targets by the type III immunity, which was to be regulated in a spatiotemporal fashion [12]. However, recent researches have revealed CRISPR-Cas10 DNases indiscriminately cleave any transcription bubbles in a close proximity both *in vivo* and *in vitro* [38]. As a result, the spatiotemporal mode of regulation of the type III immunity needs to be justified.

We recently revealed that the *Lactobacillus delbrueckii* subsp. *Bulgaricus* (Ld)Csm system mediates invader silencing by the DNase activity of the CRISPR-Cas10 system since the antiviral system does not synthesize any cOA [39]. Taking the advantage of the simplicity of this Type III immune system, mechanisms of the LdCsm immunity have been investigated in *Escherichia coli* using two-plasmid assays in which one plasmid is used for expression of LdCsm effector complexes (pCas), while the other mimics an invader (pTarget), and this revealed that the immune system is capable of degrading all cellular DNAs, including both pCas and pTarget plasmids as well as host chromosomes, suggesting that the LdCsm DNase activity protects the host cell primarily via abortive infection (ABI) [38].

While the above studies have revealed the Cas10 DNase-induced ABI mechanism, they did not yield any insight into how Cas10 DNases could function in invader clearance as suggested in previous researches [40,41]. To address this research question, we conducted two sets of experiment to examine whether the copy number of invaders could influence the turnouts of the immune response of type III Cas10 DNases. The two plasmids, pCas and pTarget, were employed again, but their copy numbers differed: one of them had the combination of a high copy pCas and a low copy pTarget and vice versa. We found that the CRISPR interference occurs in a very simple logic: the genetic element with a lower copy number is selectively eliminated from the assay system regardless of it could be pCas or pTarget. Thus, we demonstrate, for the first time, that Cas10 DNase immunity can either lead to invader clearance or induce ABI depending on the relative copy numbers of the invader and the CRISPR-carrying element in host cells.

## 2 Materials and methods

### 2.1 Bacterial strains and growth conditions

*E. coli* DH5α was the bacterial host for DNA cloning. *E. coli* BL21 (DE3) was the host for conducting plasmid interference assay. *E. coli* strains were cultured in Luria-Bertani (LB) medium. Incubation was at 37 °C, 200 rpm. If required, antibiotics were added as the following: ampicillin (Amp) at 100 μg/ml, and kanamycin (Kan) at 50 μg/ml.

### 2.2 Plasmid vectors and construction of plasmids

Two compatible *E. coli* plasmid vectors were employed for cloning in this work, including pBad and p15AIE: The former derives from pET30a and carries a ColE1 replication origin, exhibiting ca. 49 copies per cell, while the latter derives from pTRKH2 and has p15a replication origin with ca. 14 copies per cell [42], [43], [44]. In our previous works, the pCas plasmid was constructed with p15AIE (p15a-Cas-S1), carrying all *csm* genes of the III-A cas gene module and an artificial CRISPR array whereas target plasmids were based on pBad, including pColE-CTR: pTarget plasmid expressing cognate target RNAs and two reference plasmid, pColE-CK, reference plasmid lacking any target sequence and pColE-NTR expressing noncognate target RNA [38]. Since the copy number of pTarget that mimicks invader is higher than pCas that mimicks CRISPR host, this assay is named as the “high copy invader assay” (HCI assay).

Plasmids for the “low copy invader assay” (LCI assay) included the following: (a) pColE-Cas-S1, the pCas plasmid, (b) pTarget plasmid p15a-CTR as well as its reference plasmid p15a-CK and p15a-NTR. For pColE-Cas-S1 construction, ColE1-backbone (ColE1 origin plus a kanamycin-resistant gene) was amplified from pColE-CK using primers ColE-F/R whereas the expression unit of LdCsm-RNP was amplified from p15a-Cas-S1 using primers Cas-F/R. The resulting fragments were assembled using a Gibson assembly system (ABclonal Technology, Wuhan, China), giving pColE-Cas-S1. For p15a series of plasmid, the p15a-backbone (p15a origin plus an ampicillin-resistant gene) was amplified from p15a-Cas-S1 using primers p15a-F/R, while expression units of no target RNA, CTR and NTR were amplified from pColE-CK, pColE-CTR and pColE-NTR respectively using primers arab-F/R. Then, Gibson assembly was employed for generation of p15a-CK, p15a-CTR and p15a-NTR. The employed PCR primers are listed in Table S1.

### 2.3 Plasmid interference assay

In the HCI assay, *E. coli* BL21(DE3) strain carrying p15a-Cas-S1 and pColE-CTR was named as test strain (T1) to reveal the *in vivo* interference activity of LdCsm system. Another strain named reference strain (CK1) containing p15a-Cas-S1 and pColE-CK was used as a control. A third strain named NTR reference strain (NTR1) carrying p15a-Cas-S1 and pColE-NTR was for testing the influence of noncognate target RNA on the immunity. For LCI assay, test strain (T2) contained pColE-Cas-S1 and p15a-CTR, reference strain (CK2) contained pColE-Cas-S1 and p15a-CK, while NTR reference strain (NTR2) contained pColE-Cas-S1 and p15a-NTR.

Single colonies of each strain were inoculated into 20 ml LB medium containing 100 μg/ml Amp and 50 μg/ml Kan and cultured overnight at 37 °C 220 rpm. These overnight seed cultures were inoculated into 120 ml LB medium in the absence, or in the presence of Amp or Kan and inoculation was 1:100 ratio. The inoculations were allowed to grow to a mid-log phase (optical density at 600 nm (OD_600_)=0.8) at 37 °C, with shaking at 220 rpm. Each culture was then divided into 4 equal portions each of which was treated differently: (a) serving as a control, (b) addition of 0.1% l-arabinose (L-ara), (c) addition of 0.3 mM isopropyl-β-d-thiogalactopyranoside (IPTG), and (d) addition of both 0.3 mM IPTG and 0.1% l-arabinose (IPTG+*L*-ara). Samples were taken every 0.5 h/0.75 h during incubation in a total of 210 min and employed for OD_600_ measurement and for determination of colony formation units (CFU) on LB, LB+Amp, LB+Kan and LB+Amp+Kan plates. After incubation at 37 °C overnight, colonies were enumerated to get total CFU, Amp^R^ CFU and Kan^R^ CFU of the samples, from which the loss rates of pCas and pTarget plasmids of each sample were calculated.

## 3 Results

### 3.1 Experimental strategy for testing immune mechanisms of the Cas10 DNase of type III-A CRISPR system in *E. coli*

**Fig. 1:**
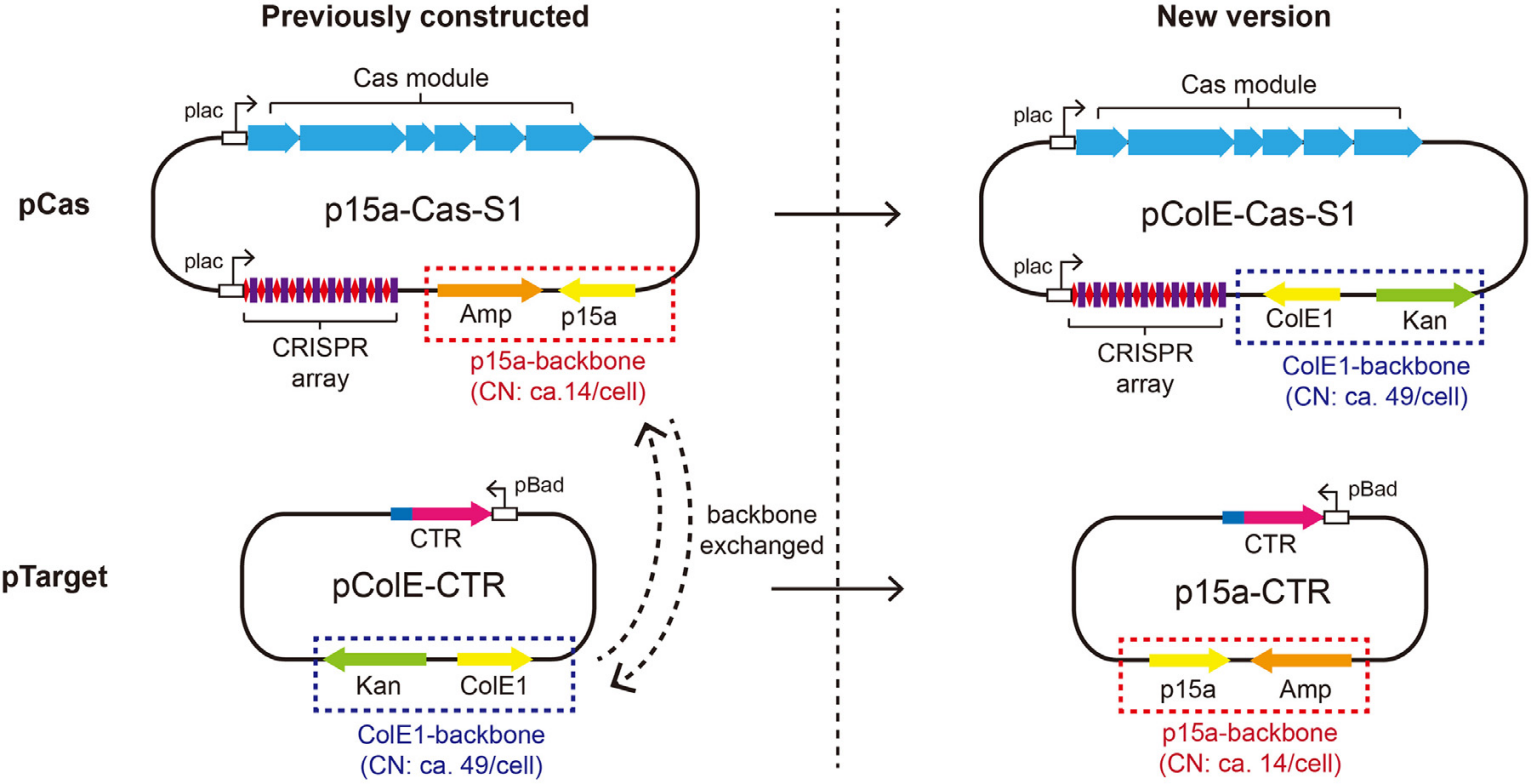
Schematic of two sets of interference plasmids used for *in vivo* assay of LdCsm immunity. Each set of interference plasmids comprises a pCas plasmid and a pTarget plasmid. pCas plasmid exhibits IPTG-inducible expression of LdCsm RNP complex. pTarget plasmid exhibits arabinose-inducible transcription of S1 RNA (CTR) targeted by LdCsm RNP complex. Simultaneous expression from both pCas plasmid and pTarget plasmid in *E. coli* host cells triggers LdCsm immunity. Previously constructed interference plasmids include p15a-Cas-S1 and pColE-CTR. Exchanging plasmid backbones between p15a-Cas-S1 and pColE-CTR produces pColE-Cas-S1 and p15a-CTR, the new set of interference plasmids. p15a-backbone carrying p15a replication origin and ampicillin-resistant gene is boxed by red dash line, whereas ColE1-backbone carrying ColE1 replication origin and kanamycin-resistant gene is boxed by blue dash line. Copy number (CN) of each plasmid backbone in *E. coli* host cell has been illustrated.

In the current literature, two plasmid interference assays are available for investigating III-A CRISPR-Cas in *E. coli*, a bacterial host that does not encode any type III CRISPR system. These include the one developed in the Terns laboratory [30] and the other one used in our group [38]. Both assays employ a two-plasmid system: pCas and pTarget (Fig. 1). In both reported assays, pCas plasmids have a relatively low copy number since they are p15a-derived plasmids that have the copy number of ca.14 per cell in average [43,44], whereas pTarget plasmids were derived from pColE1 with an average copy number of ca. 49 per cell [42,44]. The two experimental procedures differed in the following: In the first assay, selection was enforced for pCas during the induction of CRISPR immunity, by contrast, the immunity was induced in the absence of selection in the second assay. Strikingly, investigations under the pCas selection revealed that *csm6* plays an essential role in the III-A immunity [31], but interference assays in the absence of selection suggests the DNase of a III-A immunity mediates indiscriminate DNA degradation, including both plasmid and chromosome DNAs [38]. However, neither experimental approach has addressed the question of whether and how type III CRISPR-Cas systems can selectively eliminate invading DNAs from bacterial cells.

Noticeably, target RNAs were expressed from pTarget plasmids of a relatively higher copy number in both assays. As a result, the culture mimics a scenario of a high copy invader versus low copy CRISPR host and is therefore referred to as the HCI assay. However, bacterial cells could also encounter a scenario in which the copy number of invading MGEs was low. In this work, this scenario was mimicked by using a low copy plasmid for target RNA expression (the LCI assay) (Fig. 1). Both HCI and LCI experimental setups were then employed to investigate the mechanisms of Cas10 DNase immunity. These assays were started with seed cultures prepared in the presence of antibiotics whereas the actual experiments were conducted under two conditions, (a) in the absence of any genetic selection, and (b) under the selection for the pCas plasmid, and research data obtained were summarized below.

### 3.2 Onset of the Cas10 DNase immunity suppresses the growth of *E. coli* cells and induces bacterial cell lysis

**Fig. 2:**
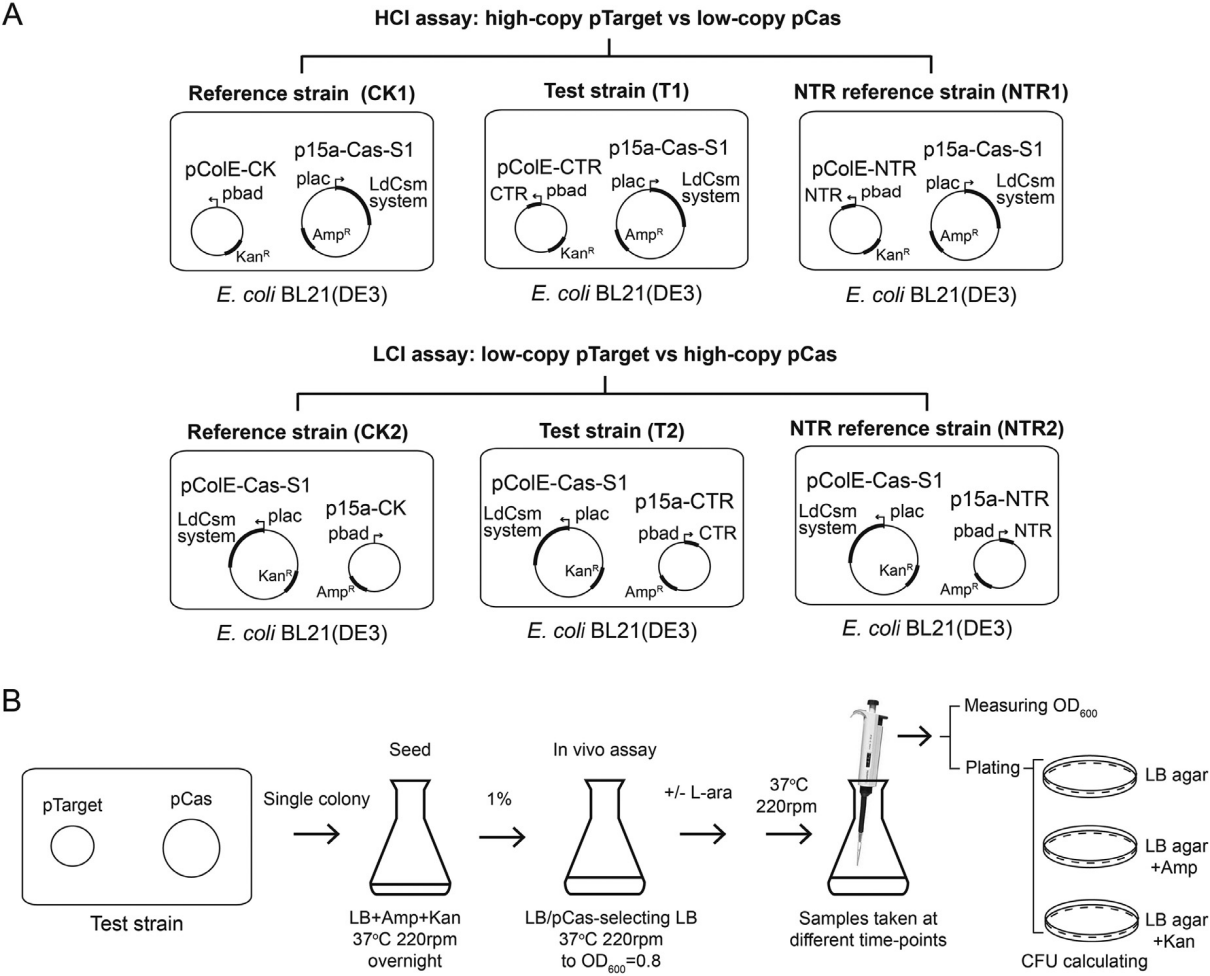
Schematic of two-plasmid assays of LdCsm DNase immunity. A Schematic of strains used in the high copy invader assay (HCI assay) and the low copy invader assay (LCI assay). Plasmids employed in the HCI assay were constructed previously [39], and those for the LCI assay were constructed in this work. Test strain in HCI assay (T1) carries p15a-Cas-S1 as the pCas and pColE-CTR as the pTarget. Its reference strain (CK1) contains p15a-Cas-S1 and pColE-CK whereas a NTR reference strain (NTR1) contains p15a-Cas-S1 and pColE-NTR. Test strain in LCI assay (T2) carries pColE-Cas-S1 and p15a-CTR. Its reference strain (CK2) contains pColE-Cas-S1 and p15a-CK while a NTR reference strain (NTR2) contains pColE-Cas-S1 and p15a-NTR. B Schematic of procedure of the *in vivo* assay. Seed cultures prepared with selection for both plasmids were divided into four portions: two were cultured in pCas-selecting LB media while the other two, with no selection. When reaching a mid-log phase (OD_600_=0.8), one in each portion was induced for target RNA synthesis while the other served as a reference.

First, we set to investigate how the LdCsm DNase could influence the culture growth and cell viability using HCI and LCI assays described above. As shown in Fig. 2A, three bacterial strains are required for each assay. Those for HCI included a reference strain (CK1), an NTR reference strain (NTR1) and a test strain (T1) all of which carried p15a-Cas-S1 (Fig. S1), the pCas plasmid but their second plasmids differed, being pColE-CK, pColE-NTR, and pColE-CTR (Fig. S1), respectively. The three strains for LCI assay were named CK2, NTR2 and T2 and they all had pColE-Cas-S1 as the pCas and the corresponding second plasmids were p15a-CK, p15a-NTR, and p15a-CTR, respectively (Fig. S2). In addition, the CTR plasmids were also called pTarget. Seed cultures were generated for each strain, which were then grown under 4 different conditions: two were cultured in the absence of any antibiotics and the other two, with the antibiotic for selecting pCas. In addition, one in each set was further supplemented with l-arabinose to induce the expression of target RNA (Fig. 2B). Cell samples were taken during the experiment and used for determination of OD_600_ and plated on different nutrient plates for determination of CFU as well as the loss rates of pCas and pTarget plasmids to assess the LdCsm immunity.

**Fig. 3:**
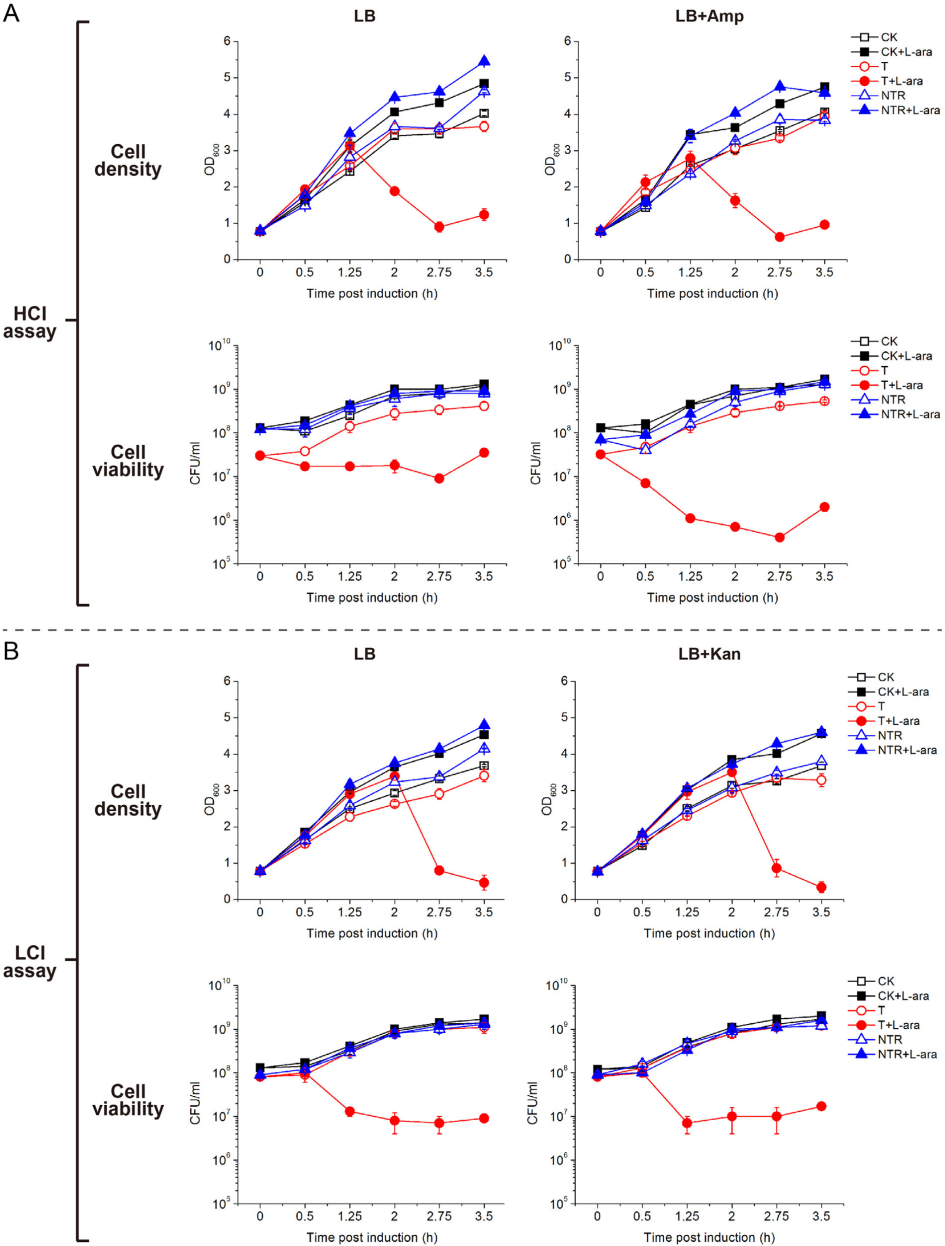
LdCsm DNase immunity impairs viability of *E. coli* cells. A Evaluation of LdCsm immunity by using growth curves in HCI assay. B Evaluation of LdCsm immunity by using growth curves in LCI assay. Test strain (T) and reference strains (CK, NTR) were cultured in LB or pCas-selecting LB medium, l-arabinose (L-ara) was added to induce the expression of target RNA. Cell samples were taken during the experiments and determined for OD_600_ values and total CFU (enumerated from LB agar plates containing no antibiotics).

In HCI assay, the two reference strains, CK1 and NTR1, showed similar growth curves under all 4 growth conditions, including the cultures with induced target RNA synthesis in comparison with their corresponding non-induced controls (Figs. 3A and S3), and these results are consistent with the previous data in which NTR is unable to elicit the LdCsm immunity [39]. For T1, however, apparent growth suppression occurred 1.25 h post induction, and the cell density reached the trough level 2.75 h post induction, with the cell density changed from OD_600_ = ca. 3.0 to < 1.0 (Figs. 3A and S3). These results suggested that majority of bacterial cells underwent cell lysis during the period of 1.25–2.75 h post induction.

Interestingly, determination of CFU revealed that the loss of cell viability occurred immediately after the induction of target RNA synthesis, i.e. > 1 h before the decrease of cell mass (Figs. 3A and S3). These results suggested that indiscriminate degradation of cellular DNAs occurs immediately upon the onset of the LdCsm immunity, yielding cell death to bacterial cells. Furthermore, for the cultures selecting for pCas, a sharp decrease of cell viability was observed for the T1 strain (Figs. 3A and S3, S4). This can be explained by the fact that the *E. coli* cells lacking p15a-Cas-S1, the pCas, can grow in LB media but will be inhibited in LB+Amp media. These results further suggested that pCas was readily lost from the T1 cells upon induction of the LdCsm immunity. Together, we clearly showed that the LdCsm DNase facilitates cell death immediately after activation of LdCsm immunity by a high level of target RNA.

We then tested if the LdCsm DNase immunity could also lead to inhibition of culture growth and to cell lysis of host cells in the LCI assay. As shown in Fig. 3B, the growth of these cultures could be divided into two phases: for 0–2 h post induction, all cultures grew similarly regardless of the induction of the LdCsm immunity and/or selection of the pCas plasmid, the second phase, the growth inhibition occurred for T2, the test strain carrying the pTarget (Figs. 3B and S5) in LCI assay, as for T1 in HCI assay (Fig. 3A). Compared to T1, the inhibitory effect in T2 was delayed for ca. 1 h (Figs. 3A, B and S3, S5). A similar delay in response to the LdCsm immunity was also observed for the loss of cell viability in T2, which was 0.5 h later than in T1 (Fig. 3A and B). On the other hand, unlike T1, cell viability of T2 in LB media was very similar to the same strain cultured in LB+Kan media selecting for pCas (Figs. 3B and S5, S6), and these results further demonstrated that pCas, the de facto CRISPR host element was stably maintained in T2 cells during the immune responses. Taken together, testing the LdCsm immunity in *E. coli* cells with the LCI assay also leads to cell death for > 99% *E. coli* cells although pCas was retained in viable cells.

Apparently, the correlation between induction of the LdCsm immunity and a strong impairment of cell viability in both HCI and LCI assays is in full agreement with our previous results where LdCsm Cas10 DNase indiscriminately cleaves plasmid and chromosome DNAs in *E. coli* cells [38]. But it remained to be investigated whether the observed cell lysis could reflect the ABI function of the LdCsm DNase and whether the DNase-based immunity could protect infected cells and eventually eliminate invading MGEs in their cells.

### 3.3 The presence of high-copy pTarget and low-copy pCas facilitates pCas clearance

We noticed that the LdCsm immunity relies on the two plasmids, pCas and pTarget, rather on the host chromosomes in these assays since LdCsm effectors are to be expressed from pCas while cognate target RNAs are to be expressed from pTarget. Thus, the loss of either plasmid would terminate the LdCsm immunity.

To investigate how a high copy pTarget could influence fate of a low copy pCas plasmid upon LdCsm immunity, HCI cell samples employed for CFU measurements were plated to reveal the presence of ampicillin-resistant pCas and kanamycin-resistant pTarget in the cells. The resulting data were then used to calculate the loss rate of these plasmids in live bacterial cells.

**Fig. 4:**
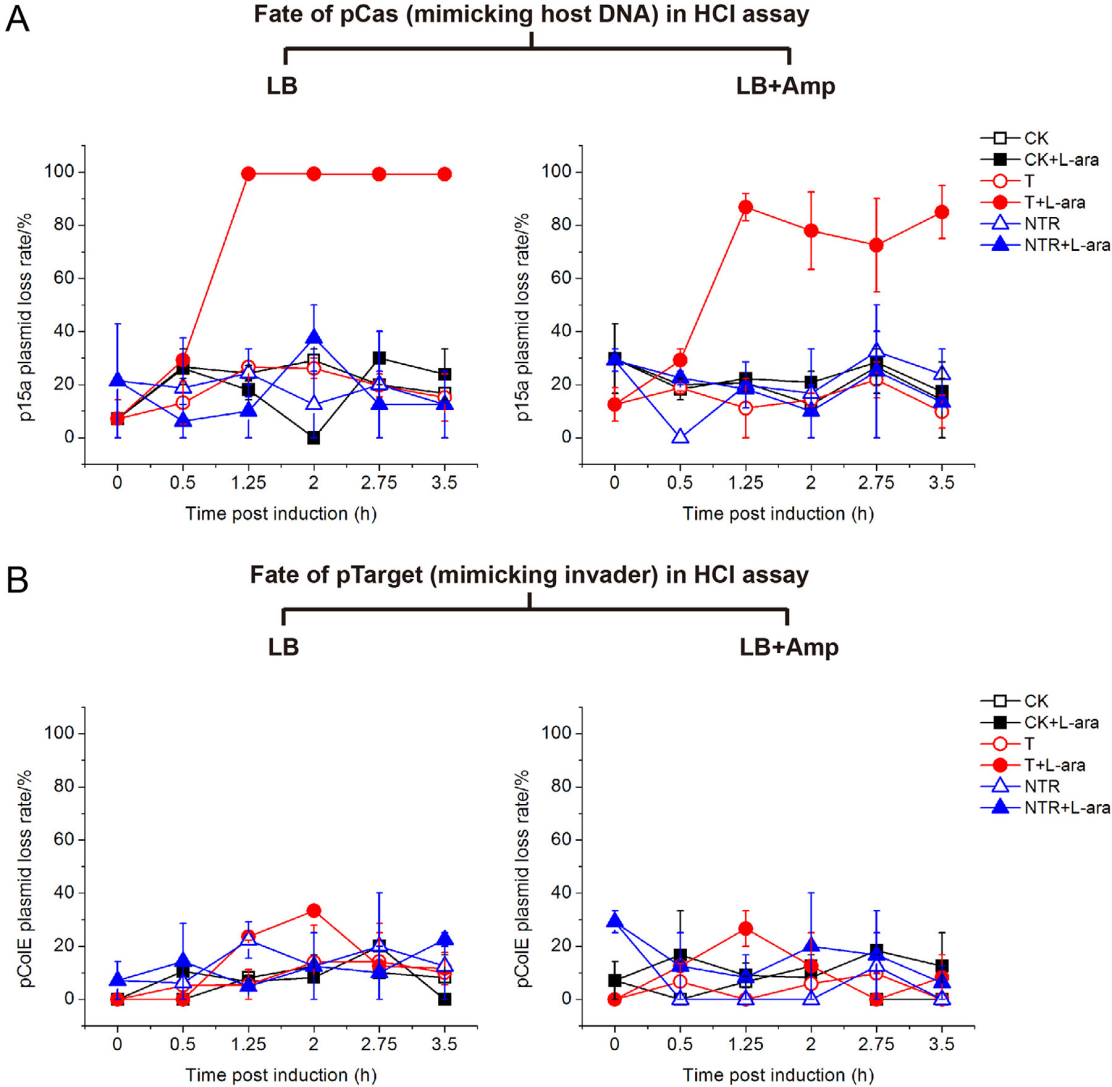
LdCsm DNase immunity causes clearance of pCas plasmid in HCI assay. A Loss rate of the host-mimicking plasmid pCas. B Loss rate of the invader-mimicking pTarget. Test strain (T) and reference strains (CK, NTR) were cultured in LB or LB+Amp medium in the presence of l-arabinose (L-ara) to induce the expression of target RNA or in the absence of l-arabinose to serve as references. Loss rates of pCas and pTarget were determined with Amp^R^ CFU, Kan^R^ CFU and total CFU of cell samples collected during the experiments.

As shown in Fig. 4A, p15a-Cas-S1 is almost completely lost from the T1 culture at 1.25 h post induction as its loss rate reached to almost 100% whereas its loss rate in the media containing Amp selecting for the same plasmid in the reference cultures was kept at ca. 80%. By contrast, plating for cells with Kan resistance revealed that pColE-CTR was stably maintained in both cultures (Figs. 4B and S3, S4). Thus, we concluded that, when copy number of pTarget is higher than pCas, pCas, the de facto CRISPR host element was eliminated by the LdCsm immunity, indicative of ABI induced by LdCsm immunity.

### 3.4 The occurrence of low-copy pTarget versus high copy pCas upon LdCsm immunity leads to pTarget clearance

**Fig. 5:**
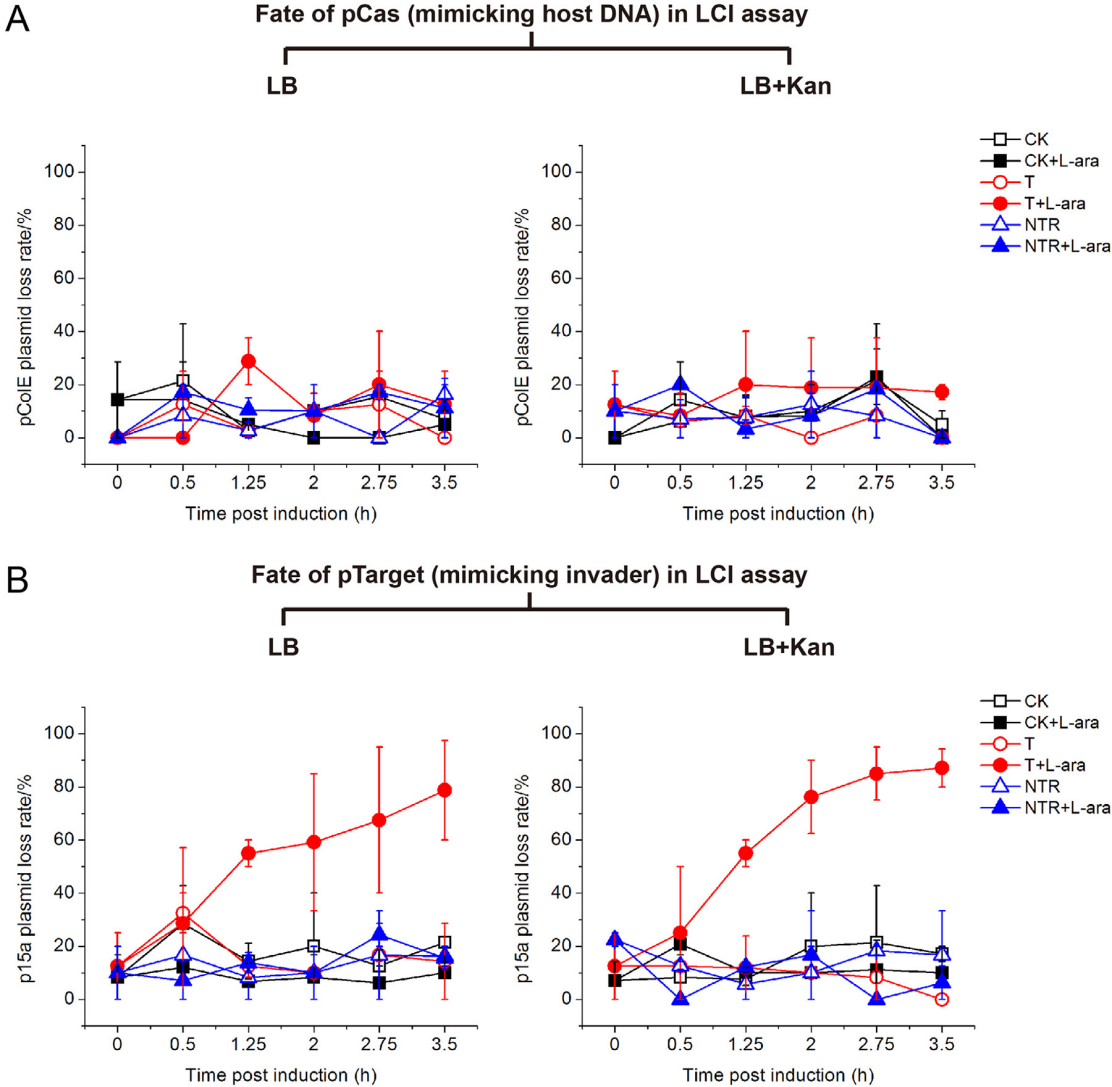
LdCsm DNase immunity causes clearance of pTarget plasmid in LCI assay. A Loss rate of the host-mimicking plasmid pCas. B Loss rate of the invader-mimicking pTarget. Test strain (T) and reference strains (CK, NTR) were cultured in LB or LB+Kan medium in the presence of l-arabinose (L-ara) to induce the expression of target RNA or in the absence of l-arabinose to serve as references. Loss rates of pCas and pTarget were were determined with Amp^R^ CFU, Kan^R^ CFU and total CFU of cell samples collected during the experiments.

We then did the same analysis for LCI cell samples. Here, the pCas plasmid is of high copy number whereas pTarget is in low copies. We found that pCas was stably maintained in all cultures (Figs. 5A and S5, S6). By contrast, pTarget and its reference plasmids were differentially maintained in the cultures of the LCI assay: while similar loss rates were determined for p15a-CK and p15a-NTR, i.e., 0∼20% under different growth conditions in the absence of immunity, the p15-CTR, a low-copy pTarget plasmid, was readily eliminated in T2, the test strain upon induction of the LdCsm DNase immunity, exhibiting loss rates of 80∼90% for 2.75∼3.5 h post induction (Figs. 5B and S5, S6). These results indicated that the LdCsm system is only capable of eliminating an invading MGE when its copy number is low.

Taken together, we have experimentally demonstrated that, upon induction of the LdCsm DNase immunity, the immune system indiscriminately targets accessible DNA substrates in all genomes present in the bacterial cell, the elimination occurs in the order of lower to higher copies of genetic elements including chromosomes until the inactivation of the immune response of the system.

## 4 Discussion

In this work, two experimental setups of plasmid interference assay have been employed to reveal the possible mechanisms of the CRISPR-Cas10 DNases immunity. Both experiments rely on testing the immunity with two plasmids in a *E. coli* host: one is characterized by a pTarget plasmid pColE-CTR with an average copy number of ca. 49 copies per cell while the other, having p15a-CTR, a plasmid of ca. 14 copies per cell, as the pTarget plasmid. Strikingly, we find that *E. coli* cells undergo cell lysis in both assays, which is demonstrated by an abrupt reduction of cell viability for > 99% after induction of the LdCsm immune response (Fig. 3). These results are congruent with our previous observations of chromosome degradation in *E. coli* host cell induced by the LdCsm immunity [38]. The *E. coli* strains employed in this assay system resemble the bacteria and archaea in which type III CRISPR systems are carried on plasmids [4], such as the Cmr variant system present in *Synechocystis* 6803 [40].

**Fig. 6:**
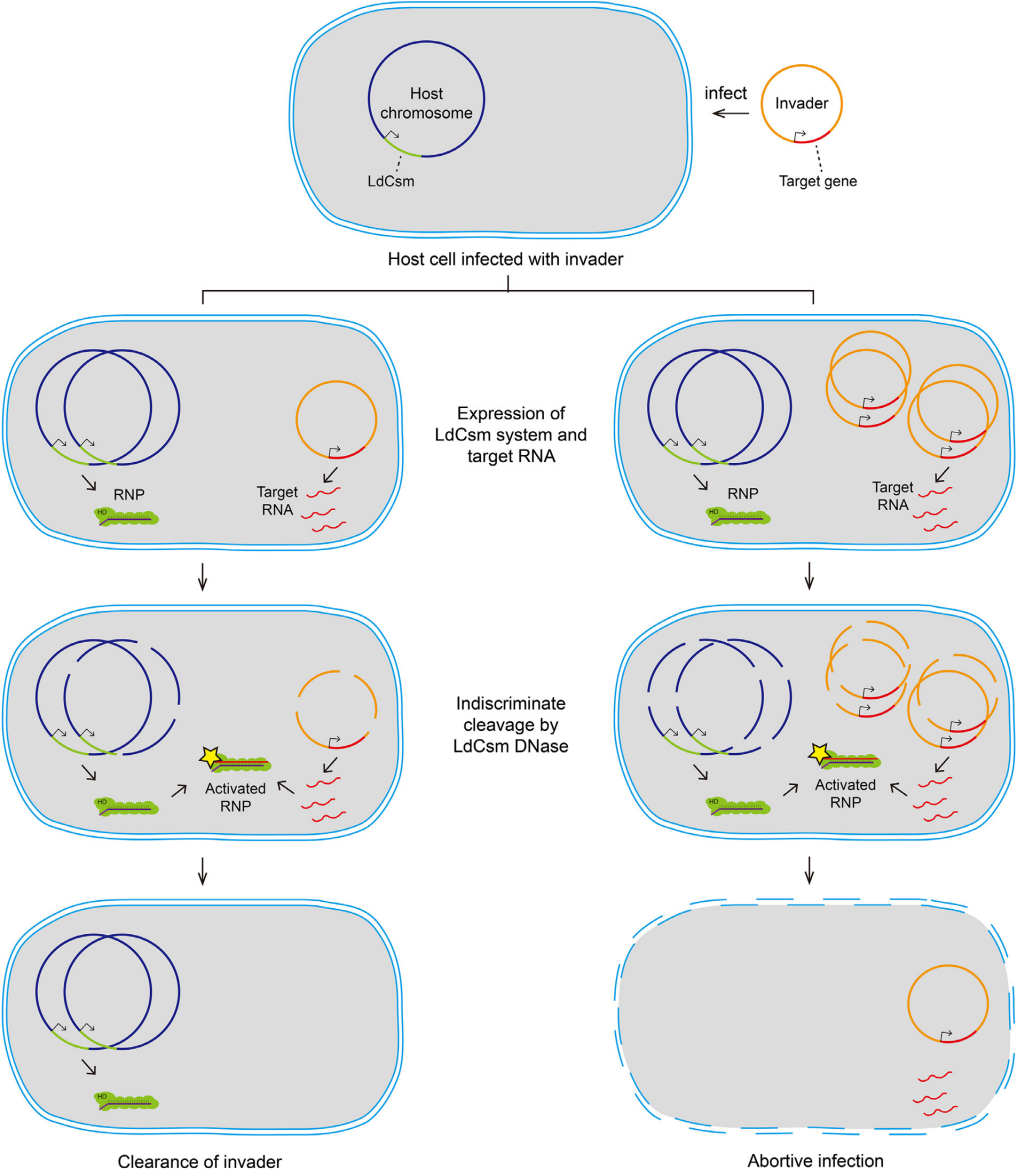
Alternative fates of microbial host cells upon activation of the LdCsm Cas10 DNase immunity. When a host cell carrying a type III CRISPR system is invaded by a MGE, two scenarios can occur: (a) the MGE is to be targeted at a stage of low copies, and (b) the MGE is to be targeted in a high copy stage. The first scenario leads to invader clearance while the second one yields abortive infection.

However, most type III CRISPR systems are encoded on the chromosomes of bacteria or archaea [4]. In this scenario, pCas represents the de facto CRISPR host element whereas pTarget plasmids mimic the invading MGEs. Based on this definition, the results obtained with the two plasmid interference assays can be readily applied to these chromosome-encoded CRISPR-Cas10 systems. Upon the activation of the LdCsm immunity, the loss of the pCas plasmid should be regarded as the occurrence of ABI whereas the loss of the pTarget plasmid meant a successful defense event. In conclusion, we have documented in this work that the LdCsm DNase immunity can either induce ABI or invader clearance depending on the copy number of invaders although the DNase exhibits indiscriminate DNA cleavage activity (Fig. 6).

In these two-plasmid assays, the *E. coli* chromosomes should be the most vulnerable genome to the LdCsm DNase compared with the two plasmids since their copy numbers are predicted as ca. 1:3:9 (chromosome/p15a/pColE plasmids) in the *E. coli* cells in these experiments. Theoretically, none of *E. coli* cells could survive the LdCsm DNase immunity if the ratio would be kept constant. However, unlike *E. coli* chromosomes that segregate equally during cell division, pColE and p15a plasmids are randomly divided upon cell division since they do not encode any segregation/partition systems. As a result, while most daughter cells would receive high copies of both plasmids, a small proportion of cells could only receive one or a few copies of p15a plasmid, which would make p15a plasmids even more vulnerable to the LdCsm DNase than the bacterial chromosome in the cell. This could account for the observation that it is always the p15a plasmid that was eliminated by the LdCsm DNase, regardless whether it functions as a host-mimicking or invader-mimicking plasmid (Figs. 4A and 5B).

Our finding also fits very well with results obtained from plasmid interference assays conducted with protospacer plasmids in native hosts where plasmids, which are in a low copy upon transformation are readily eliminated by the CRISPR systems, as reported for several type III CRISPR-Cas10 DNases [33,35,36,39,40,45].

Our model can also be employed to explain the phenomenon in which type III Cas10 DNase immunity is capable of eliminating invading MGEs at early stages of invasion but requires the signal transduction pathway to curb the replication of MGEs as well as their gene expression in late stages. In most characterized type III CRISPR-Cas10 systems, cOA synthesis by Cas10 Palm domain and cOA-activated indiscriminate RNA cleavage by CARF domain effectors, such as Csm6/Csx1, have been reported to play a major role in anti-plasmid/virus immunity [31], [32], [33], whereas their CRISPR-Cas10 DNase appears to be relatively weak. As our assays have confirmed that strong Cas10 DNase would induce cell lysis, it makes sense that more type III systems have not evolved to get as strong Cas10 DNase as LdCsm system to avoid killing the host cell during immune process. It is thus evolutionarily advantageous to have a relatively weak DNA cleavage but a strong cOA synthesis activity. In the hypothesis by Rostol and Marraffini et al. [36], the cOA-activated Csm6 degrades both host and invading transcripts in type III-A immunity and caused cell dormancy, which would gain the time required for invader clearance by CRISPR-Cas10 DNases, and the two-plasmid assay reported here can be employed to test this hypothesis.

In addition, our research has also experimentally demonstrated another level of regulation of the LdCsm DNase immunity, i.e., the relative expression levels of LdCsm effectors vs. CTR RNAs. Compared to the LCI assay, cell lysis in the HCI assay occurred 0.5∼0.75 h earlier under l-arabinose induction (Fig. 3). Even in the absence of the induction, HCI test strains showed slight growth suppression than the reference strain whereas LCI test strains did not. Moreover, loss of p15a plasmid in the HCI assay was more dramatic than in the LCI assay (Figs. 4A and 5B). These results indicated that the LdCsm DNase activity in the HCI strains was higher than in the LCI strains. Indeed, the expression of CTR RNAs from a high copy pTarget and LdCsm effectors from a low copy pCas yields a higher ratio of CTR RNAs vs. LdCsm that expands the time window of the RNA-activated LdCsm DNase in HCI test cells. By contrast, CTR RNA expression from a low copy pTarget and LdCsm effector synthesis from a high copy pCas should yield a low ratio of CTR RNAs vs. LdCsm that reduces the time window of the activated LdCsm DNase in LCI test cells. Apparently, elegant designs of two-plasmid assays are required to investigate the influence of both plasmid copy numbers and expression levels of type III CRISPR effectors and their target RNAs, etc. on ABI and invader clearance, which would yield further insights into the mechanisms of how the DNA cleavage and cOA signaling pathway of CRISPR-Cas10 systems cooperate in antiviral defense and eventually fine tuning of the immunity to best fit the physiology of different bacterial hosts.

## CRediT authorship contribution statement

Z.Y. designed the experiments, carried out most of the experiments, analyzed the data, prepared figures and drafted the manuscript. Y.Z., J.X. carried out part of the experiments. Q.S. designed the experiments, analyzed the research data, wrote and revised the manuscript with the help from Z.Y. and Y.Z.

## Declaration of Competing Interest

The authors declare the following financial interests/personal relationships which may be considered as potential competing interests: Given his role as Executive Editor, Dr. Qunxin She had no involvement in the peer-review of this article and has no access to information regarding its peer-review. Full responsibility for the editorial process for this article was delegated to Dr. Ming Li.

## Appendix Supplementary materials

Image, application 1
