# Supplementary material for: The influence of the copy number of invader on the fate of bacterial host cells in the antiviral defense by CRISPR-Cas10 DNases
Source: Eng Microbiol. 2023 Jun 24;3(4):100102. doi: 10.1016/j.engmic.2023.100102 (PMC11610955; doi:10.1016/j.engmic.2023.100102)
Supplement: Supplementary file 1 [file mmc1.pdf]

This file includes:

Figure S1 to Figure S6

Table S1

Supplemental figures

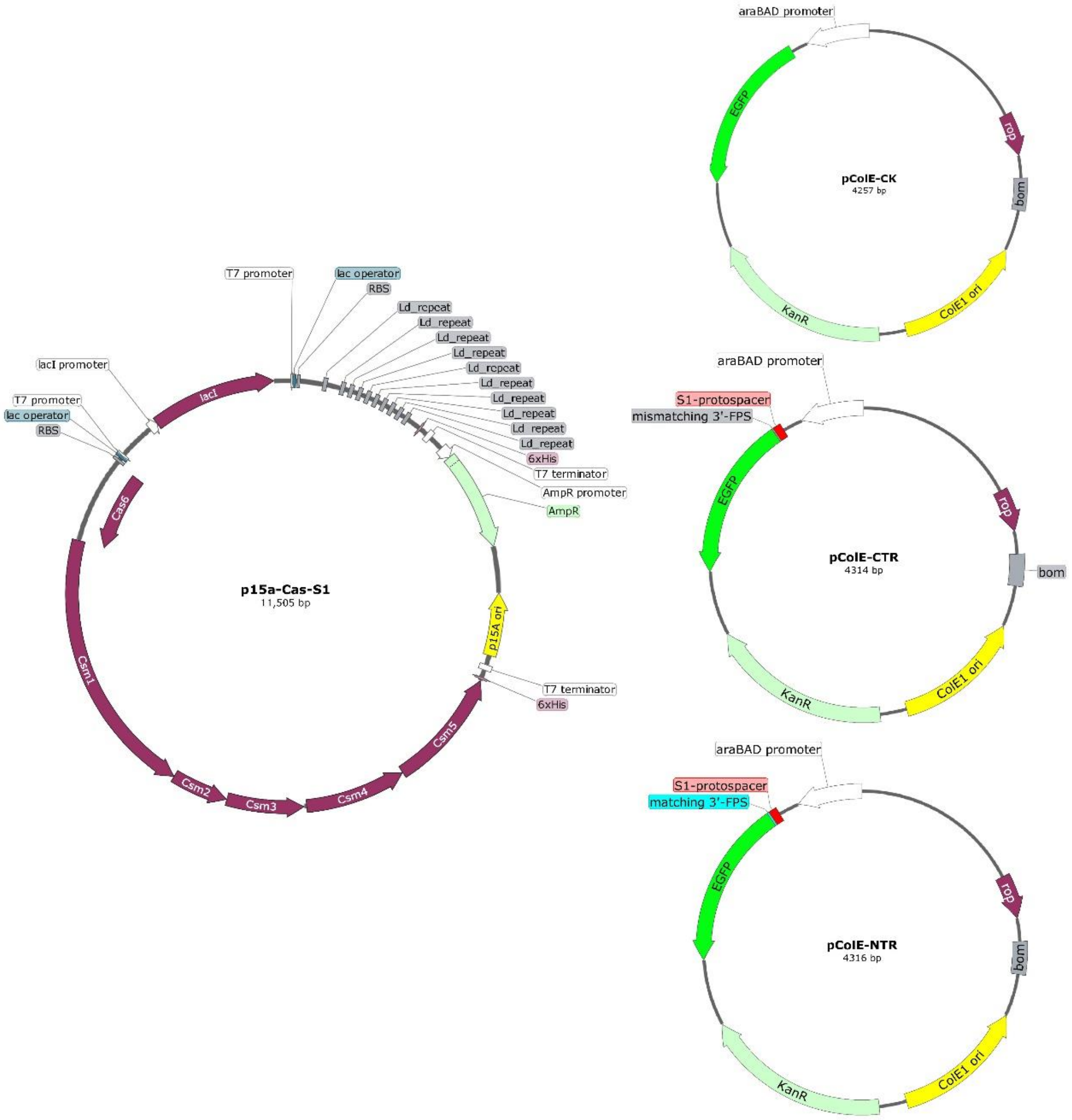

Figure S1. pCas and pTarget used in HTA assay (constructed by Lin et al in 2021). Related to Figure 1, Figure2.

pCas plasmid p15a-Cas-S1 is composed of a LdCsm-expressing cassette (Ld *cas* cassette and a mini CRISPR array of S1 spacer, under control of *lac* promoter) and a p15a-backbone (p15a replication origin plus an ampicillin-resistant gene). pTarget plasmid pColE-CTR is composed of a cognate target RNA (CTR) expressing cassette (under control of *araBad* promoter) and a ColE1 backbone (ColE1 origin plus a kanamycin-resistant gene). Non-cognate target RNA expressing plasmid pColE-NTR has a non-cognate target RNA expressing cassette. Reference plasmid pColE-CK does not carry target sequence.

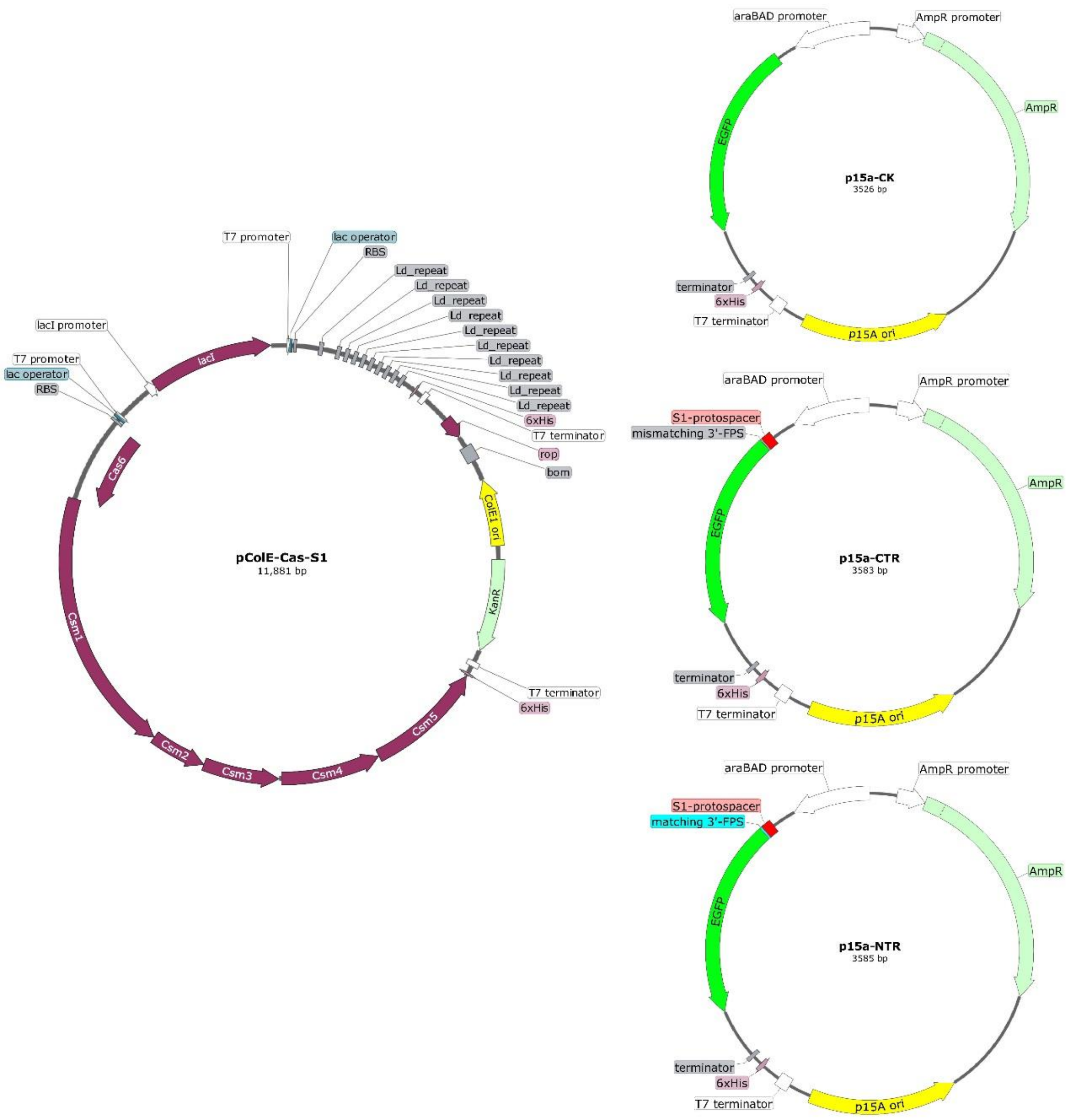

Figure S2. pCas and pTarget used for LTA assay (constructed in this work). Related to Figure 1, Figure 2.

pCas plasmid pColE-Cas-S1 is composed of a LdCsm-expressing cassette (Ld *cas* cassette and a mini CRISPR array of S1 spacer, under control of T7 promotor and *lac* operator) and a ColE1-backbone (ColE1 origin plus a kanamycin-resistant gene). pTarget plasmid p15a-CTR is composed of a cognate target RNA (CTR) expressing cassette (under control of *araBad* promoter) and a p15a backbone (p15a origin plus an ampicillin-resistant gene). Non-cognate target RNA expressing plasmid p15a-NTR has a non-cognate target RNA expressing cassette. Reference plasmid p15a-CK does not carry target sequence.

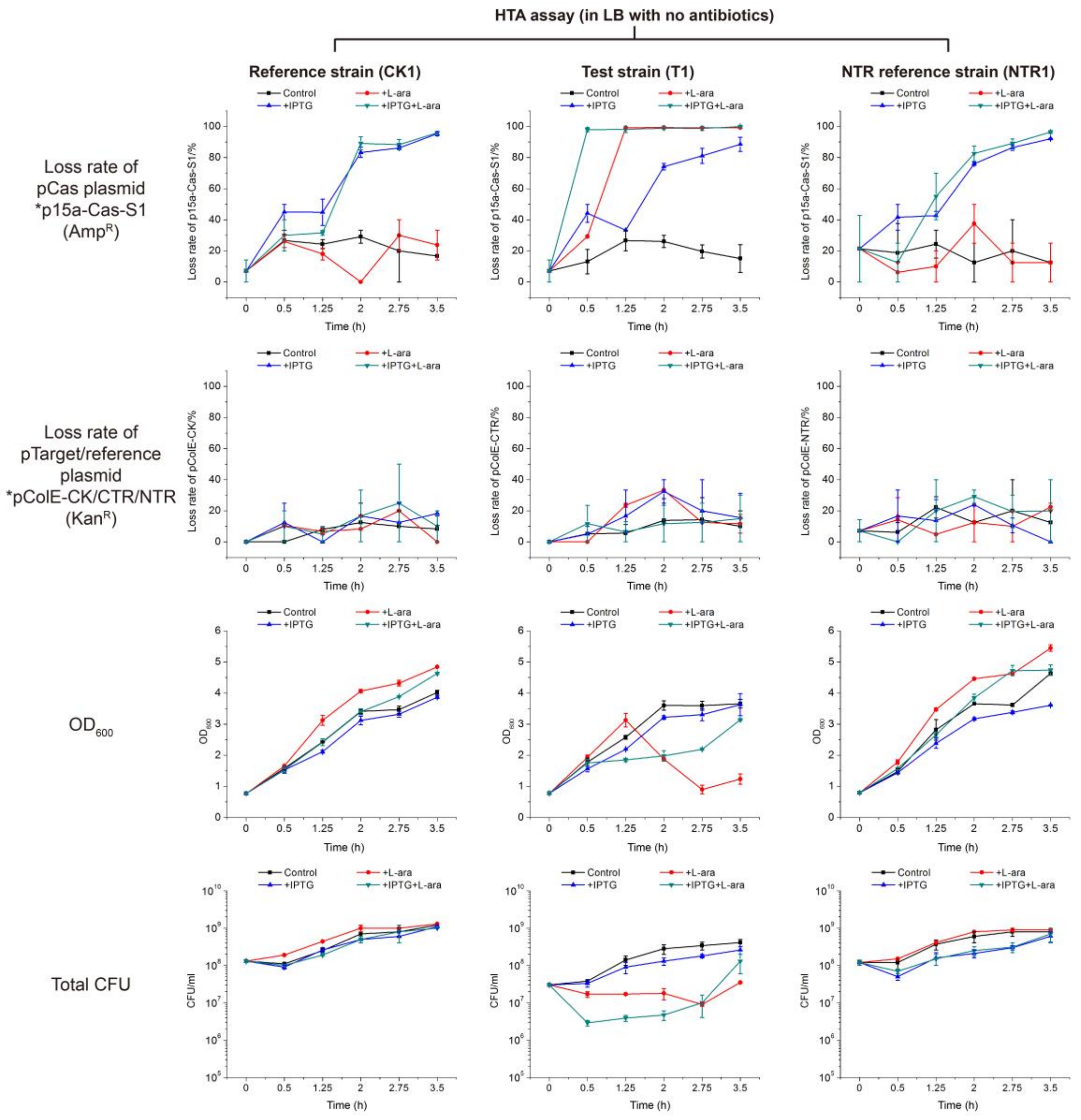

Figure S3. Evaluation of LdCsm DNase interference activity by HTA assay. Related to Figure 3A, Figure 4.

Test strain (T) and reference strains (CK, NTR) were cultured in LB media to a mid-log phase (OD_{600}=0.8). Then these cultures were each divided into four equal portions and induced by no inducers (control), L-arabinose (L-ara), ITPG or IPTG+L-ara respectively. OD_{600}, total CFU, and loss rate of pCas and pTarget (revealed by determination of AmpR CFU, KanR CFU and total CFU) of these strains at different time-points post induction were measured.

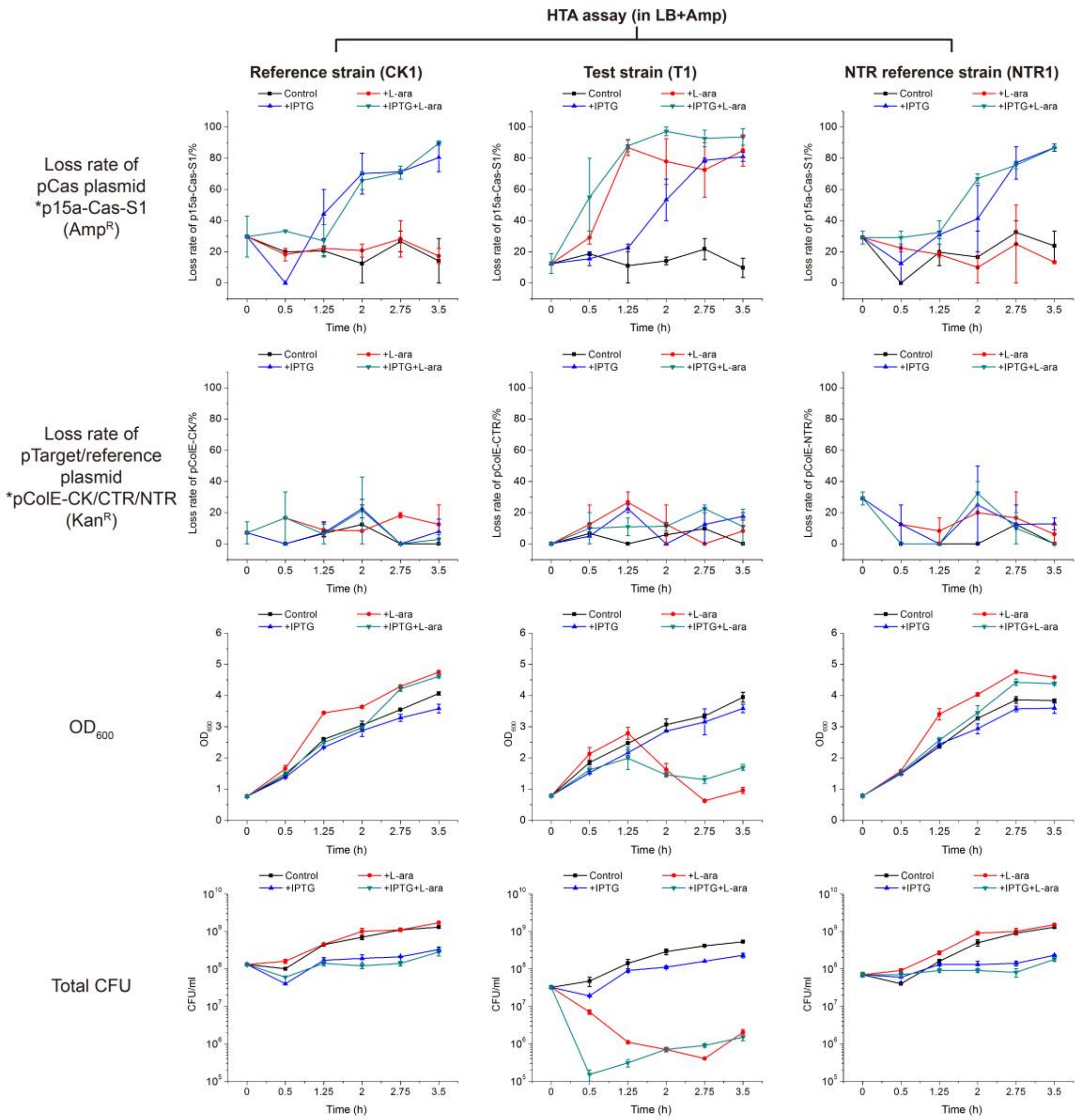

Figure S4. Evaluation of LdCsm DNase interference activity by HTA assay with pCas under selection. Related to Figure 3A, Figure 4.

Test strain (T) and reference strains (CK, NTR) were cultured in LB+Amp (pCas-selecting) media to a mid-log phase (OD_{600}=0.8). Then these cultures were each divided into four equal portions and induced by no inducers (control), L-arabinose (L-ara), ITPG or IPTG+L-ara respectively. OD_{600}, total CFU, and loss rate of pCas and pTarget (revealed by determination of AmpR CFU, KanR CFU and total CFU) of these strains at different time-points post induction were measured.

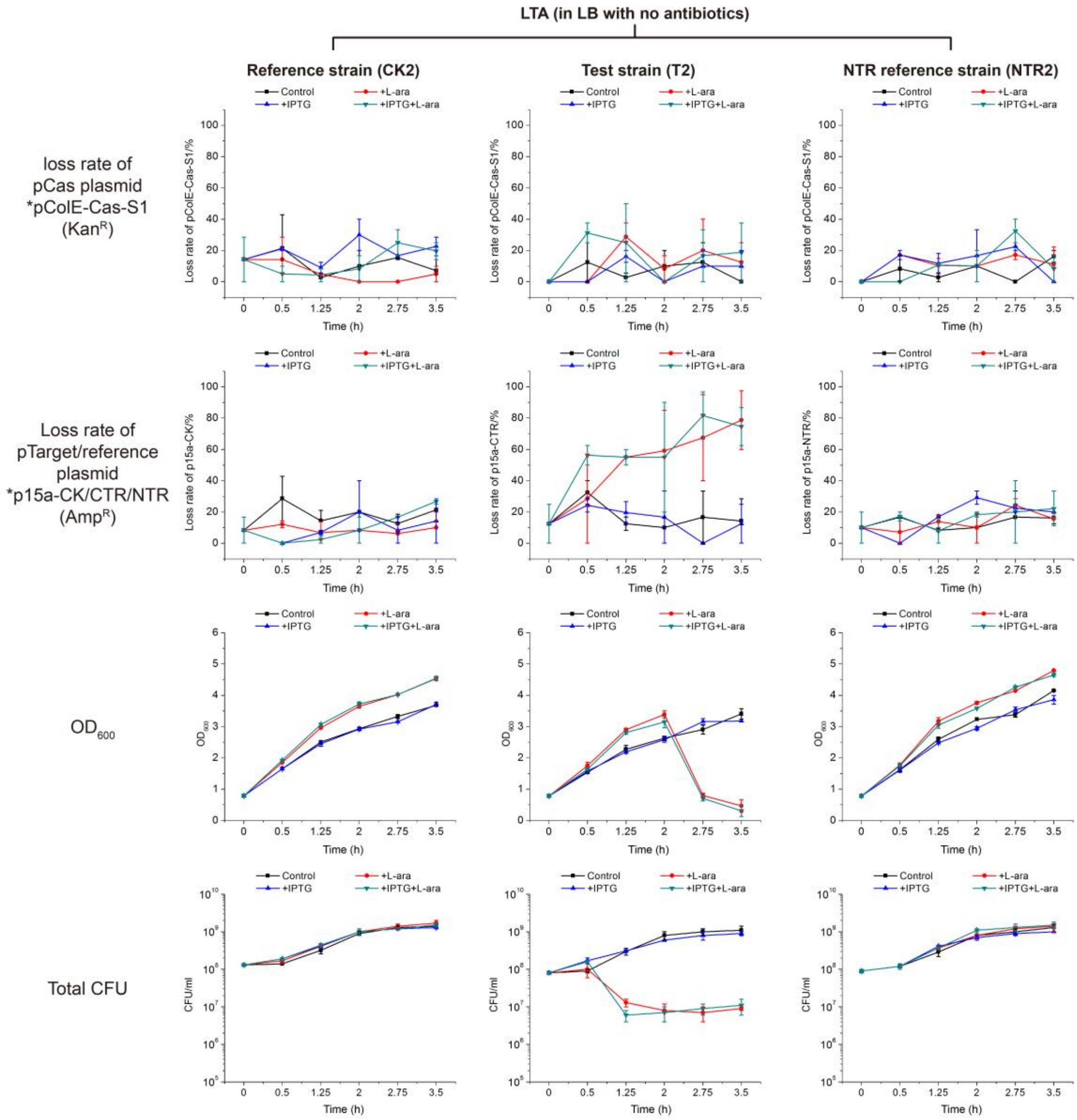

Figure S5. Evaluation of LdCsm DNase interference activity by LTA assay. Related to Figure 3B, Figure 5.

Test strain (T) and reference strains (CK, NTR) were cultured in LB media to a mid-log phase (OD_{600}=0.8). Then these cultures were each divided into four equal portions and induced by no inducers (control), L-arabinose (L-ara), ITPG or IPTG+L-ara respectively. OD_{600}, total CFU, and loss rate of pCas and pTarget (revealed by determination of AmpR CFU, KanR CFU and total CFU) of these strains at different time-points post induction were measured.

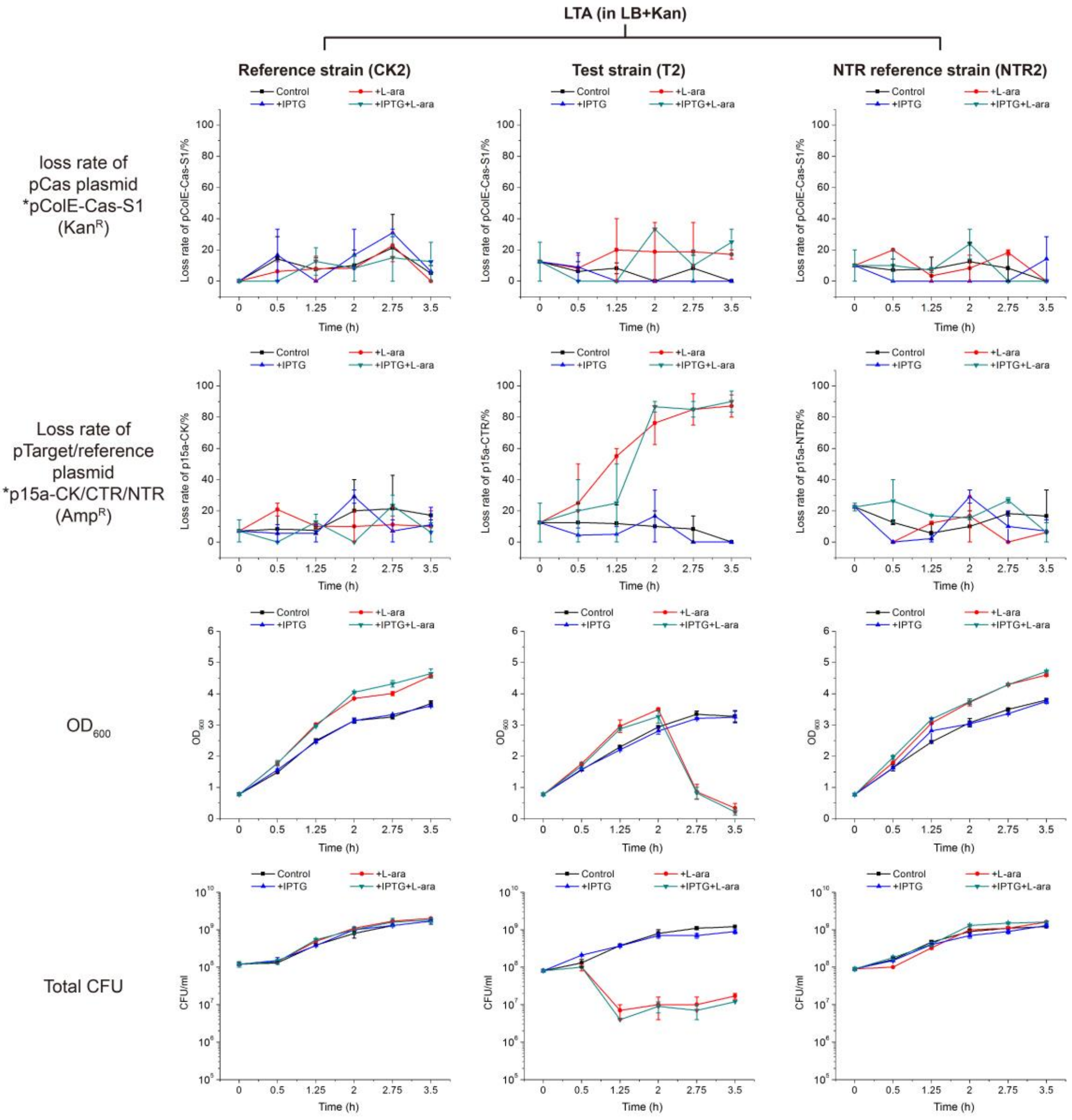

Figure S6. Evaluation of LdCsm DNase interference activity by LTA assay with pCas under selection. Related to Figure 3B, Figure 5.

Test strain (T) and reference strains (CK, NTR) were cultured in LB+Kan (pCas-selecting) media to a mid-log phase (OD_{600}=0.8). Then these cultures were each divided into four equal portions and induced by no inducers (control), L-arabinose (L-ara), ITPG or IPTG+L-ara respectively. OD_{600}, total CFU, and loss rate of pCas and pTarget (revealed by determination of AmpR CFU, KanR CFU and total CFU) of these strains at different time-points post induction were measured.

Supplemental table

Table S1 ssDNA primers used in this study

Primers	Sequence (5'-3')	Description
Cas-F	TCCGCGCACATTTCCCCGAACCGCCCTGCAGATCCGGA	pColE-Cas-S1 construction
Cas-R	GTAGGTGTTCCACAGGGTAGAATAGGCGTATCACGAGGCCCT	pColE-Cas-S1 construction
ColE-F	GGCCTCGTGATACGCCTATTCTACCCTGTGGAACACCTACATC	pColE-Cas-S1 construction
ColE-R	TCCGGATCTGCAGGGCGGTTCGGGGAAATGTGCGCGGA	pColE-Cas-S1 construction
arab-F	GCTAGCAGAAACCAATTGTCCATATTGCATCAGAC	p15a-CK/CTR/NTR construction
arab-R	GAGCTCAAAAAAAAGATTTTCAACAGAACCGTTTCTACTCAATGA	p15a-CK/CTR/NTR construction
p15a-F	GATGCAATATGGACAATTGGTTTCTGCTAGCGACGAAAGGGCCT	p15a-CK/CTR/NTR construction
p15a-R	CGGTTCTGTTGAAAATCTTTTTTTTGAGCTCCGTCGACAAGCTTG	p15a-CK/CTR/NTR construction
